# Unveiling the mystery of scale dependence of surface roughness of natural rock joints

**DOI:** 10.1038/s41598-022-04935-3

**Published:** 2022-01-19

**Authors:** Yingchun Li, Hongwei Yang, Shengyue Sun

**Affiliations:** 1 State Key Laboratory of Coastal and Offshore Engineering, Dalian University of Technology, Dalian, 116024 China; 2 School of Civil Engineering, Sun Yat-sen University, Guangdong, 528406 China

**Keywords:** Solid Earth sciences, Engineering

## Abstract

Scale dependence of surface roughness of natural rock joints has long been an outstanding issue in rock mechanics. Controversial results were reported by various studies; however, the nature of scale dependency and the underlying mechanism for the conflicting observations remain unclear. Rock joints at different scales characterise two-order asperities, namely, waviness and unevenness; thus understanding how the individual roughness of waviness and unevenness vary as the joint size increases from the laboratory-scale to the large-scale is crucial for revealing the scale effect mystery. Here we digitise three natural granite joint surfaces with the same dimension of 1000 mm × 1000 mm through a high-resolution, three-dimensional scanner. Waviness and unevenness of each rock joint surface are quantitatively separated by selecting an appropriate sampling interval. The respective fractal dimensions of waviness and unevenness of joint surfaces sized from 100 mm × 100 mm to 1000 mm × 1000 mm are estimated through an improved roughness-length method. We find that the fractal dimensions of two-order roughness are scale-dependent but without generalised trends. The stationarity threshold beyond which the scale-dependency of roughness vanishes is absent for all the three joint samples, suggesting that the roughness of natural rock joints be assessed at the specific scale of the rock mass in-situ. We reveal that previous controversial results regarding scale effect are likely due to the composition of the roughness scaling of waviness and unevenness. Thus, accurate stability analysis of rock-engineering projects should consider separate contributions of multi-order asperities across scales to the strength and deformation of jointed rock masses.

## Introduction

Rock mass stability relies heavily on the mechanical properties of rock joints. When a joint is present, the rock mass strength is substantially reduced since the rock block can easily slide along the joint surface. The slide or shear behaviour of a rock joint is strongly affected by the surface roughness that inherently exists in broad sizes from millimetres to kilometres. Therefore, roughness quantification at different scales is critical for predicting the shear behaviour of rock joints in the field.

The shear behaviour of a rock joint varies as the joint size changes, which is termed scale effect. The scale effect of the joint shear behaviour mainly results from the variation of the naturally formed surface roughness. Bandis et al.^1^ documented a positive scale effect (*We follow the definition of Bandis et al.*^1^ *that decreased surface roughness with increasing scale is called positive scale effect and vice versa. The reason for this clarification is that some studies described the scale effect on the contrary way*^2,3^), i.e., both the surface roughness and joint shear strength decreased as the joint size increased. However, conflicting observations including negative and no scale effect have also been reported^2,4–8^. Moreover, some studies claimed that the scale-dependence of joint surface roughness is restricted to a certain size, i.e., a stationarity threshold and the roughness descriptors remain nearly unvaried for the sample size higher than this limit^9,10^. Due to these controversial results, the extent and nature of scale effect still eludes explanation.

**Figure 1:**
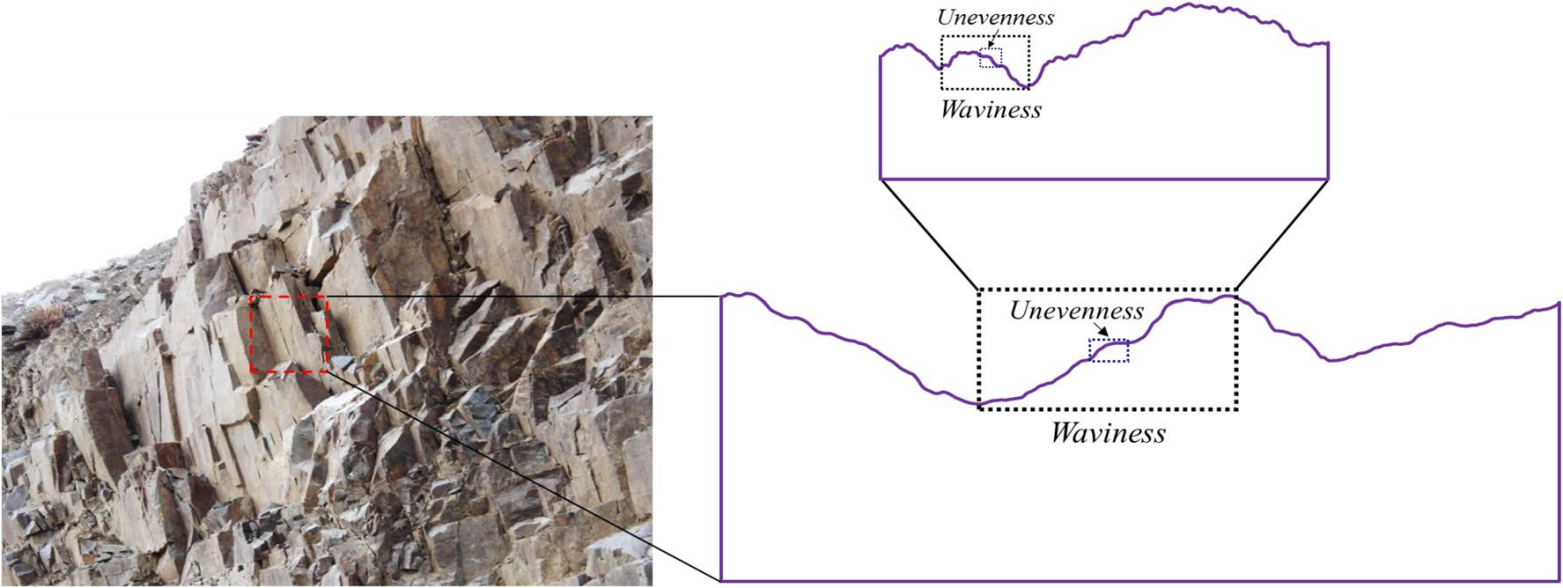
Rock joint surfaces exhibit waviness and unevenness at different scales.

To reveal the scale dependence of the shear behaviour of a rock joint, how the surface roughness controls the shear process should be first understood. For a sawtooth-shaped joint, the asperities override each other without noticeable damage, provided that the normal stress is low. When the normal stress grows appreciably, the asperities undergo considerable degradation^11,12^. Natural rock joints are rough with irregular asperities, and their roughness degree are commonly rated by *JRC* (Joint Roughness Coefficient)^13^. Joint dilation and degradation are then quantified through the variation of *JRC*^13–16^. Nevertheless, these studies mathematically characterise the variation of roughness degree but neglects the mechanical involvements of roughness at different orders^17–19^. A natural rock joint at each scale possesses two-order roughness, i.e., waviness and unevenness^20^ (Fig. 1). Under a low normal stress, waviness and unevenness mutually govern the shear behaviour of a rock joint. When the normal stress ascends pronouncedly, waviness dominates dilatancy and shear resistance since unevenness is easily sheared-off. Therefore, the changes of waviness and unevenness at various scales are key to unveil the mystery of the scale effect of joint shear behaviour. It also has been reported that the fluid flow in rock fractures depends highly on the distribution of waviness and unevenness^21,22^. Nevertheless, existing investigations have rarely separated a whole joint surface into waviness and unevenness and examined corresponding roughness scaling^20^.

In this study, we investigate the scale dependence of two-order roughness of natural rock joints through fractal characterisation. The surface morphology of three large-scale granite joints sized 1000 mm × 1000 mm are digitised by a high-resolution, 3D optical scanner. The waviness and unevenness of a natural joint surface is quantitatively decomposed, which enables accurate estimation of the fractal dimension of each-order roughness by a modified roughness-length method. No apparent stationarity threshold is observed although the fractal dimensions of waviness and unevenness are scale-dependent.

## Data acquisition

**Figure 2:**
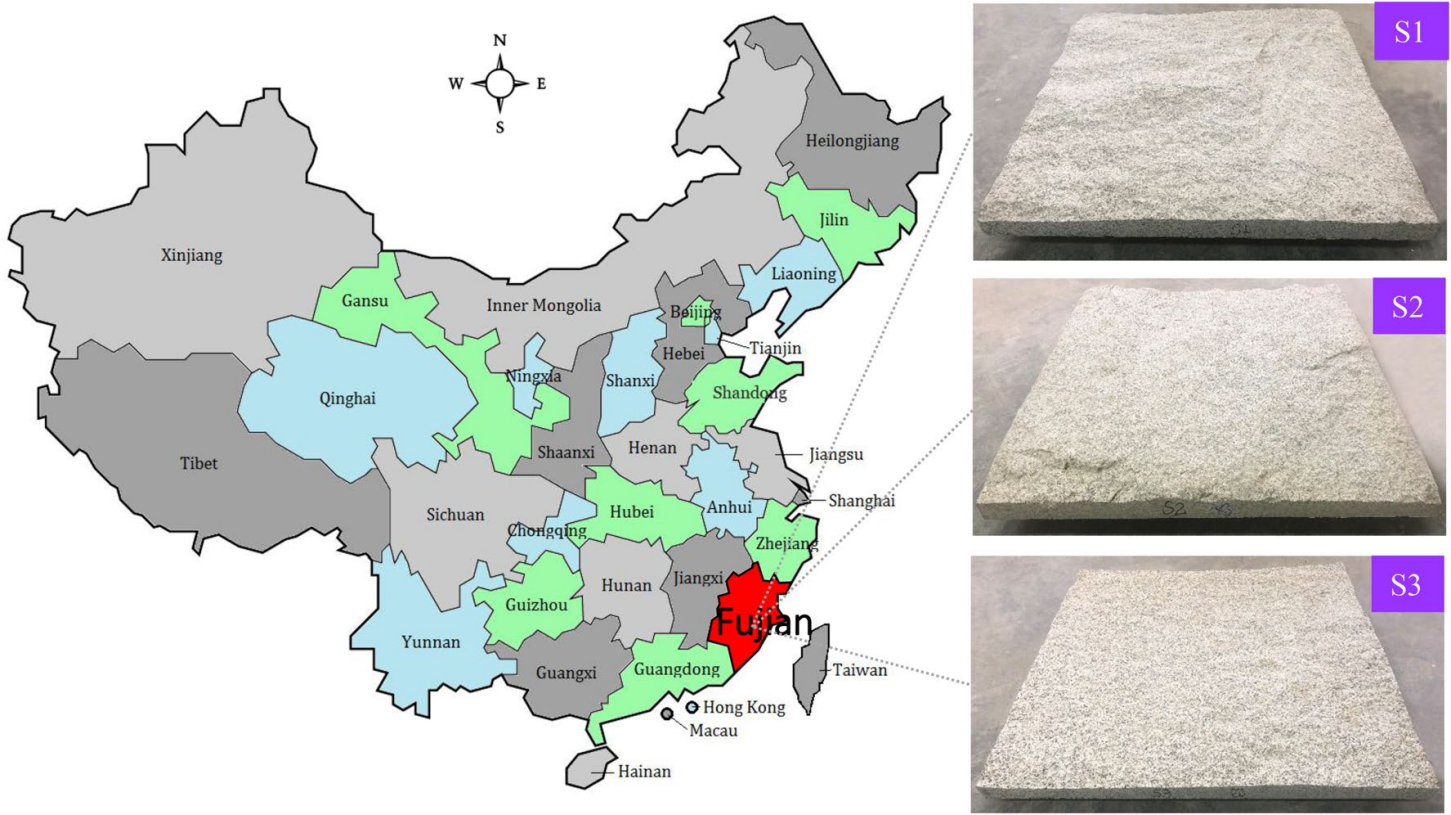
Three 1000 mm × 1000 mm rock joints sourced from Fujian province, China.

With the aid of a high-resolution optical scanning system, CREAFORM Metra Scan 3D-system, we digitised the surfaces of three granite joints (denoted S1, S2, and S3, respectively) in the same size of 1000 mm × 1000 mm at three different resolutions, i.e., 0.5 mm, 1.0 mm, and 2.0 mm. The three natural, large-scale granite rock joints were ordered from a vendor specialising in rock-related business. The joint samples were sourced from the mountainous region, Fujian province located in the southeast of China (Fig. 2). The surfaces of the three joint samples were slightly gray and the grain size was 0.5 mm to 1.0 mm.

**Figure 3:**
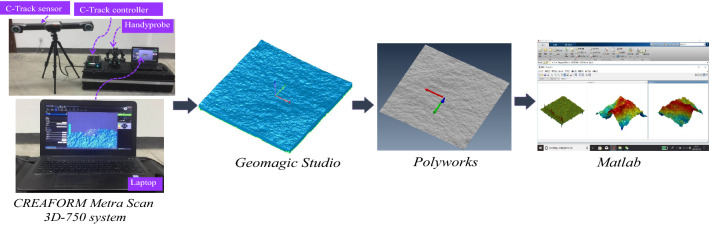
Surface digitisation of a 1000 mm × 1000 mm rock joint.

The powerful scanning system can digitise and reconstruct an object up to several metres with the highest resolution of 0.05 mm. It has four core components, including the C-Track senor to capture the object under scanning, the C-Track controller to cache the digitised data, the Handyprobe to manually scan, and a laptop to store the digitised data and display (Fig. 3). Over scanning, the rock joints surfaces were digitised region by region to ensure that there was no void left. Then the digitised data was imported to the image processing software, Geomagic Studio for coordinating. Another image processing software, Polyworks processed the morphological information into readable files by Matlab in which following analysis was conducted (Fig. 3).

## Fractal dimensions of waviness and unevenness

### Roughness separation

**Figure 4:**
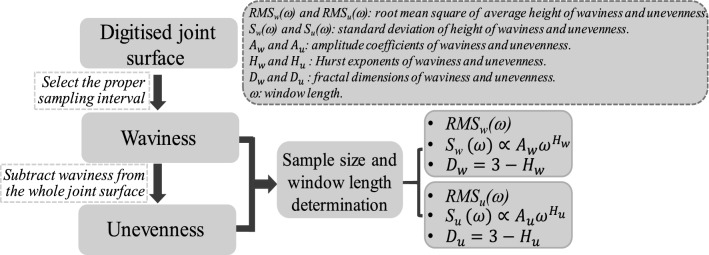
Flowchart to decompose roughness and fractal dimension estimation.

Waviness and unevenness are asperities exhibiting in two different orders, indicating that they are separable by selecting an appropriate sampling interval. Several approaches have been employed to separate waviness from the total surface, including the triangular prism area method (TPM)^23^, surface area method (SAM)^24^, Fourier series transformation^25^, and wavelet analysis^19,21^. The Fourier series and wavelet analysis approach simulates the original joint profile by the composition of sinusoidal curves and thus some details may be lost over the reconstruction and decomposition process. Here, we use a sampling interval selection approach to realize the separation of waviness from the total surface, similar to the signal processing approach extensively used in the disciplines of electronics and telecommunication. We first determine the waviness of a digitised joint surface by stepwisely increasing the sampling interval at the increment of the measurement resolution until the displayed surface after filtering best matches the shape of the joint surface (Fig. 4). The highest matching quality is determined by visual examination through trial-and-error^21^. The unevenness is then acquired by subtracting the waviness from the whole joint surface. To enable the subtraction, the data points of the waviness are interpolated to ensure that the data arrays of the waviness and the whole joint surface are in the same size. Compared to the TPM and SAM that require cumbrous calculation to obtain the relationship between the total area of numerous digitized elements and different sampling intervals, the sampling interval selection approach is simpler and more convenient to conduct.

**Figure 5:**
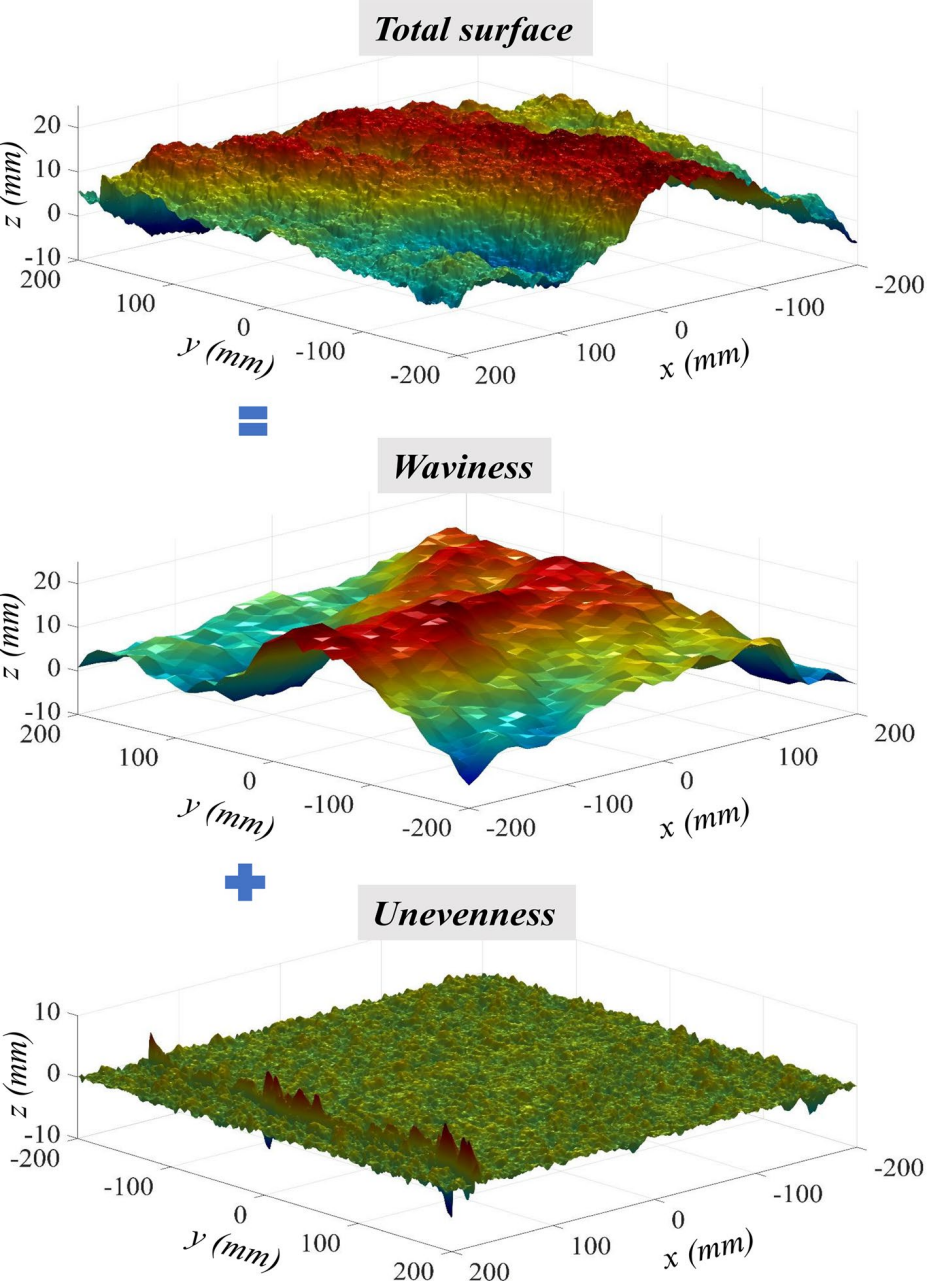
Separation of a rock joint surface into waviness and unevenness.

Following the above procedures, we decomposed the three joint surfaces at different measurement resolutions into waviness and unevenness. Figure 5 demonstrates the separated waviness and unevenness of the joint sample S3 at the resolution of 0.5 mm. During the decomposition, we surprisingly found that the sampling intervals at which the waviness of the three joint samples were extracted have the same value of 10 mm. The consistency of the sampling interval possibly due to that the three granite joint samples are sourced from the same origin where the geological processes creating rock joints are similar.

### Improved roughness-length method

Many indictors have been available to quantify the joint surface roughness^13,26–28^, among which the fractal approach has been extensively employed to characterise the surface roughness of rock joints at various scales^2,9,20,29–35^. The attraction of the fractal method lies in its nature that it can predict the scaling behaviour, i.e., the relationship between surface geometry observed at various scales, which is consistent with the requirement of the current study that aims to reveal the roughness variation of joint surfaces at different scales. Moreover, the fractal dimension is easy to calculate and can serve as a quantitative roughness indicator for both two dimensional joint profiles and three dimensional joint surfaces^9,10^. A natural or artificial fractal object exhibits a geometrically repeating pattern at each scale^36^. If the replication is exactly the same at every scale, it is termed self-similar in which all directions are scaled with the same magnifications. In contrast, the fractal is self-affine with different magnifications in differing directions^37^. Natural rock joints are commonly self-affine since the rigorous requirements of self-similarity are scarcely satisfied^9,31,38–40^.

Several approaches have been proposed to estimate the fractal dimension of a joint profile/surface^28^. It is well recognized that the fractal dimension of self-affine natural joints can be accurately estimated by the roughness-length method^9,31,38–40^. Moreover, the roughness-length method is developed based on clear physical interpretation, namely the relationship between the standard deviation of the asperity height (the average asperity height representing the geometrical property of a natural joint) and the joint size^41^. Specifically, for a self-affine joint surface, the standard deviation of the asperity height ($S_H(\omega )$) is associated with the window length ($\omega$) through the following power function^41^:

1 $$\begin{aligned} S_H(\omega )=A\cdot \omega ^H \end{aligned}$$

Double-logarithmise Eq. (1), we have:

2 $$\begin{aligned} \ln \left[ S_H(\omega )\right] =\ln A+H\ln \omega \end{aligned}$$

where *A* and *H* are amplitude coefficient and Hurst exponent, respectively. The values of *A* and *H* are measurable from the ln $[S_H(\omega )]$-$\ln \omega$ relationship. When $\omega = 1$, $S_H(\omega ) = A$, i.e., *A* characterises how the joint amplitude amplifies at a particular scale. The Hurst exponent (*H*) represents the degree at which the joint surface flattens with increasing sizes^42^. showed that the Hurst exponent (*H*) is related to fractal dimension (*D*) through:

3 $$\begin{aligned} H=E-D \end{aligned}$$

where *E* is the Euclidean dimension (two for a profile and three for a surface). A joint surface with a low value of the Hurst exponent owns a high fractal dimension.

**Figure 6:**
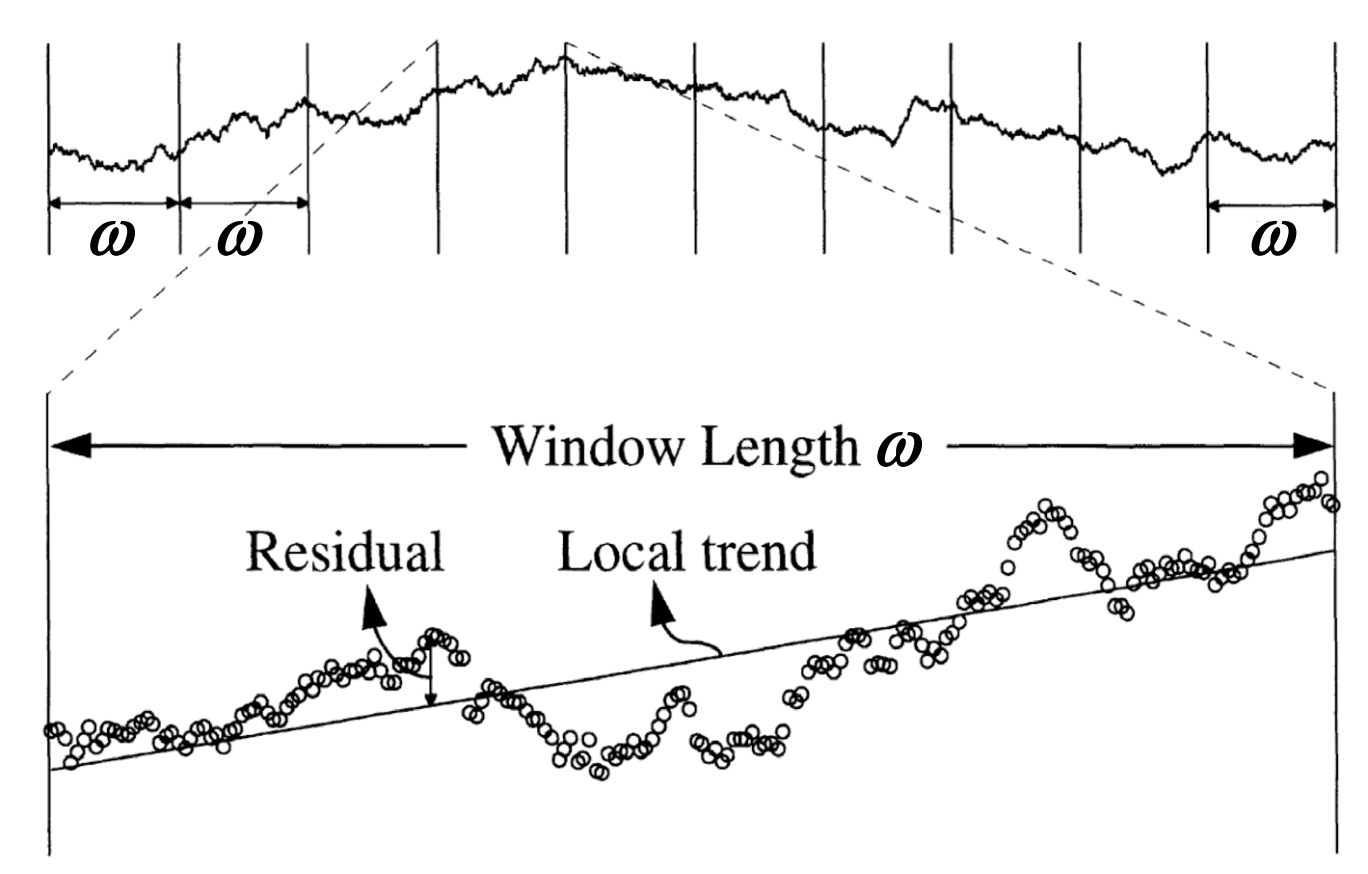
Illustration of the original roughness-length method (after Kulatilake and Um^31^). $\omega$ represents the window length.

Malinverno^41^ reported that the longer wavelengths representing the trend of the joint surface should be excluded for accurately estimating the roughness in the sampled windows. The standard deviation of the asperity height ($S_H(\omega )$) was quantified as the *RMS* (root mean square) of the surface height residuals on a local trend linearly fitting the measurement data in the window length ($\omega$) (Fig. 6), i.e.:

4 $$\begin{aligned} S_H(\omega )=RMS(\omega )=\frac{1}{n_\omega }\sum _{i_1}^{n_\omega } \sqrt{\frac{1}{m_i-2}\sum _{j\in \omega _i}(z_i-\bar{z})^2} \end{aligned}$$

where $n_\omega$, $m_i$, $z_j$, and $\bar{z}$ respectively represent the total number of windows, the number of points included in each window, the residuals on the trend, and the mean residual in the window $\omega _i$.

**Figure 7:**
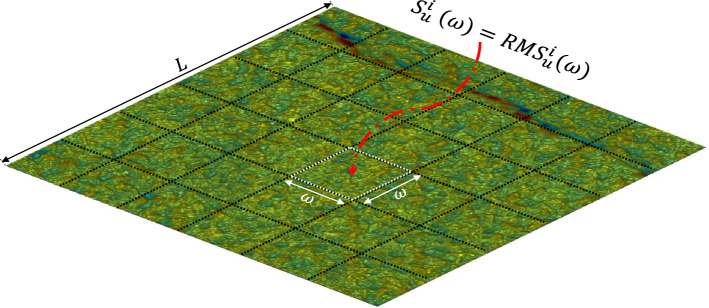
Window length ($\omega$) selection of a separated unevenness. *L* and $\omega$ are the sample length and the window length, respectively. $S^i_u(\omega )$ and $RMS^i_u(\omega )$ are the standard deviation and the root mean square of the height of unevenness in a window, respectively.

According to Eq. (2), the fractal dimensions of waviness and unevenness ($D_w$ and $D_u$, respectively) are estimated by:

5a $$\begin{aligned} \ln \left[ S_w(\omega )\right]= & {} \ln A_w+H_w\ln \omega \end{aligned}$$

5b $$\begin{aligned} \ln \left[ S_u(\omega )\right]= & {} \ln A_u+H_u\ln \omega \end{aligned}$$

where $S_w(\omega )$ and $S_u(\omega )$ represent the standard deviations of the heights of waviness and unevenness, respectively. In the approach of Malinverno^41^, waviness was removed from the joint surface roughness, and a local trend that was a linear correlation of the waviness was used as the reference line to calculate the standard deviation of asperity heights ($S_H(\omega )$) using the *RMS* of the surface height residuals [see Eq. (4)]. That is to say, the original roughness-length method only used the roughness with small wavelength for fractal dimension calculation. In this study, since waviness and unevenness are separated, the standard deviations of heights of waviness and unevenness ($S_w(\omega )$ and $S_u(\omega )$) should be individually assessed based on the original definition of *RMS* without excluding the local trend of a profile, i.e.:

6a $$\begin{aligned} S_w(\omega )=& \, {} RMS_w(\omega )=\frac{1}{n^\omega _w}\sum _{i_1}^{n^\omega _w} \sqrt{\frac{1}{m^i_w}\sum _{j\in \omega ^i_w}\left( h^i_w-\bar{h_w}\right) ^2} \end{aligned}$$

6b $$\begin{aligned} S_u(\omega )= & \, {} RMS_u(\omega )=\frac{1}{n^\omega _u}\sum _{i_1}^{n^\omega _u} \sqrt{\frac{1}{m^i_u}\sum _{j\in \omega ^i_u}\left( h^i_u-\bar{h_u}\right) ^2} \end{aligned}$$

where $RMS_w(\omega )$ and $RMS_u(\omega )$ denote the root mean squares of the asperity heights of waviness and unevenness, respectively; $n^\omega _w$ and $n^\omega _u$ are the total number of windows of waviness and unevenness, respectively; $m^i_w$ and $m^i_u$ represent the number of points included in each window of waviness and unevenness, respectively; $\omega ^i_w$ and $\omega ^i_u$ are the *i*th windows of waviness and unevenness, respectively; $h^i_w$ and $h^i_u$ are the heights of waviness and unevenness, respectively; and $\bar{h_w}$ and $\bar{h_u}$ are the average heights of waviness and unevenness, respectively (Fig. 7). The window length ($\omega$) had a maximum value of 20% of the sample length and a minimum value containing at least ten data points^41^. Additionally, the sample length (*L*) was dividable by the corresponding window length ($\omega$).

## Results and analysis

**Figure 8:**
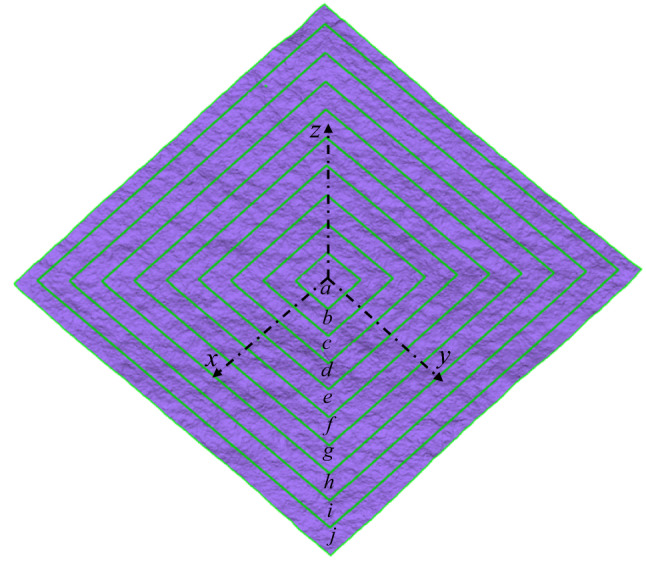
Square joint samples in different sizes from 100 mm × 100 mm to 1000 mm × 1000 mm chosen from the central part of a rock joint surface.

**Figure 9:**
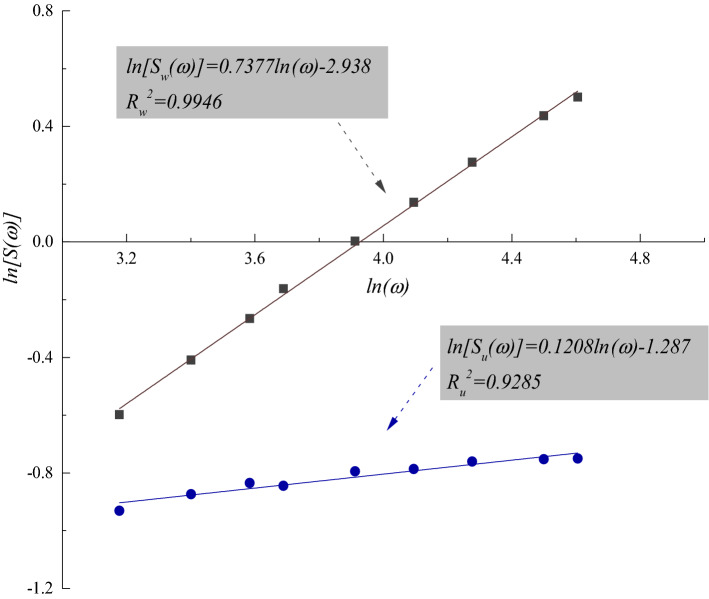
Double-logarithmic relationship between standard deviations of heights of two-order roughness ($S_w(\omega )$ and $S_u(\omega )$, respectively) and window length ($\omega$) of the sample size of $900 \mathrm \, mm \times 900 \mathrm \, mm$ of joint sample S1. $D_w$ and $D_u$ denote the fractal dimensions of waviness and unevenness, respectively. $R^2_w$ and $R^2_u$ are the coefficients of determination in estimating the fractal dimensions of waviness and unevenness, respectively.

To disclose the scale dependence of the two-order roughness, we estimated the respective fractal dimensions of waviness and unevenness of the joint surfaces sized from 100 mm × 100 mm to 1000 mm × 1000 mm at an interval of 100 mm × 100 mm through Eqs. (5) and (6). The square joint surfaces were sampled from the central part of a rock joint surface outwards (Fig. 8). Figure 9 illustrates the double-logarithmic relationships between the standard deviation of heights of two-order roughness ($S_w(\omega )$ and $S_u(\omega )$, respectively) and window length ($\omega$) of the joint sample S1 with a sample size of 900 mm × 900 mm. Tables 1, 2 and 3 detail the calculated fractal parameters including fractal dimensions ($D_w$ and $D_u$, respectively) and amplitude coefficients ($A_w$ and $A_u$, respectively) of waviness and unevenness of the three joint samples in sample sizes from 100 mm × 100 mm to 1000 mm × 1000 mm under three different resolutions. All the coefficients of determination ($R^2_w$ and $R^2_u$) are satisfactorily high, demonstrating the applicability and capability of Eqs. (5) and (6).

**Table 1:** Fractal dimensions of waviness and unevenness of rock joint sample S1 at varying sample sizes.

Resolution (mm)	Fractal estimation	Sample size ($\mathrm mm \times mm$)									
		$100 \times 100$	$200 \times 200$	$300 \times 300$	$400 \times 400$	$500 \times 500$	$600 \times 600$	$700 \times 700$	$800 \times 800$	$900 \times 900$	$1000 \times 1000$
0.5	$D_w$	2.1520	2.1617	2.1865	2.1585	2.2316	2.2106	2.2273	2.2316	2.2623	2.2505
	$A_w$	− 3.373	− 3.237	− 3.074	− 3.093	− 2.909	− 3.028	− 3.006	− 3.020	− 2.938	− 2.973
	$R^2_w$	0.9985	0.9992	0.9979	0.9989	0.9949	0.9977	0.9972	0.9982	0.9946	0.9937
	$D_u$	2.6782	2.7546	2.7019	2.7190	2.7312	2.8284	2.7409	2.8942	2.8792	2.8776
	$A_u$	− 2.169	− 1.875	− 1.948	− 1.822	− 1.836	− 1.409	− 1.796	− 1.210	− 1.287	− 1.149
	$R^2_u$	0.9955	0.9421	0.9582	0.9323	0.9455	0.9338	0.9265	0.9251	0.9285	0.9674
1.0	$D_w$	2.2594	2.2145	2.2341	2.1845	2.2678	2.2544	2.2641	2.2713	2.3010	2.3042
	$A_w$	− 2.410	− 2.445	− 2.389	− 2.391	− 2.207	− 2.304	− 2.309	− 2.316	− 2.251	− 2.216
	$R^2_w$	1.0000	0.9997	0.9987	0.9987	0.9985	0.9979	0.9986	0.9996	0.9967	0.9945
	$D_u$	2.6913	2.7627	2.8697	2.8753	2.6819	2.8871	2.8554	2.9061	2.8976	2.9208
	$A_u$	− 2.002	− 1.743	− 1.303	− 1.163	− 1.161	− 1.171	− 1.277	− 1.132	− 1.191	− 0.962
	$R^2_w$	0.9954	0.9493	0.9368	0.9181	0.9258	0.9325	0.9848	0.9238	0.9171	0.9273
2.0	$D_w$	2.4097	2.1913	2.2624	2.1844	2.3209	2.2796	2.2936	2.2872	2.3509	2.3505
	$A_w$	− 1.573	− 1.954	− 1.710	− 1.818	− 1.535	− 1.687	− 1.696	− 1.749	− 1.590	− 1.564
	$R^2_w$	1.0000	0.9975	0.9920	0.9963	0.9960	0.9953	0.9972	0.9990	0.9956	0.9903
	$D_u$	2.7348	2.8312	2.8836	2.8415	2.8879	2.9206	2.9379	2.9366	2.9460	2.935
	$A_u$	− 1.630	− 1.541	− 1.295	− 1.298	− 1.215	− 1.128	− 1.099	− 1.118	− 1.101	− 0.941
	$R^2_u$	1.0000	1.0000	0.9113	0.9124	0.9118	0.9783	0.9756	0.9918	0.9531	0.9326

$D_w$ and $D_u$ are fractal dimensions of waviness and unevenness, respectively. $A_w$ and $A_u$ are the coefficients during linear correlations for estimating $D_w$ and $D_u$, respectively. $R^2_w$ and $R^2_u$ represent the coefficients of determination during linear correlations for estimating $D_w$ and $D_u$, respectively.

**Table 2:** Fractal dimensions of waviness and unevenness of rock joint sample S2 at varying sample sizes.

Resolution (mm)	Fractal estimation	Sample size ($\mathrm mm \times mm$)									
		$100 \times 100$	$200 \times 200$	$300 \times 300$	$400 \times 400$	$500 \times 500$	$600 \times 600$	$700 \times 700$	$800 \times 800$	$900 \times 900$	$1000 \times 1000$
0.5	$D_w$	2.1663	2.2253	2.2264	2.2501	2.2412	2.2200	2.2330	2.2508	2.447	2.2242
	$A_w$	− 3.248	− 3.114	− 3.091	− 3.004	− 2.988	− 3.070	− 3.029	− 2.978	− 2.972	− 3.059
	$R^2_w$	0.9978	0.9959	0.9952	0.9944	0.9960	0.9985	0.9974	0.9968	0.9962	0.9976
	$D_u$	2.6690	2.7391	2.6969	2.7573	2.6501	2.7402	2.7288	2.7443	2.7335	2.7737
	$A_u$	− 2.142	− 1.900	− 1.972	− 1.763	− 2.171	− 1.857	− 1.881	− 1.852	− 1.855	− 1.579
	$R^2_u$	0.9898	0.9393	0.9000	0.9745	0.9928	0.9175	0.9305	0.9193	0.9119	0.9395
1.0	$D_w$	2.2448	2.3214	2.2892	2.3257	2.2884	2.2586	2.2754	2.2944	2.2852	2.2664
	$A_w$	− 2.471	− 2.252	− 2.317	− 2.212	− 2.259	− 2.364	− 2.314	− 2.269	− 2.271	− 2.335
	$R^2_w$	1.0000	0.9978	0.9950	0.9941	0.9993	0.9993	0.9989	0.9991	0.9973	0.9988
	$D_u$	2.6250	2.6325	2.8642	2.9069	2.6473	2.9132	2.8751	2.9210	2.9117	2.9195
	$A_u$	− 2.062	− 2.057	− 1.315	− 1.199	− 1.989	− 1.180	− 1.281	− 1.148	− 1.153	− 0.976
	$R^2_w$	1.0000	0.9977	0.9363	0.9761	0.9962	0.9496	0.9585	0.9941	0.9230	0.9277
2.0	$D_w$	2.2194	2.3634	2.3395	2.3433	2.3378	2.2842	2.2892	2.3141	2.3313	2.2840
	$A_w$	− 2.037	− 1.627	− 1.666	− 1.646	− 1.645	− 1.759	− 1.764	− 1.701	− 1.617	− 1.762
	$R^2_w$	1.0000	0.9911	0.9906	0.9930	0.9969	0.9992	0.9978	0.9982	0.9958	0.9975
	$D_u$	2.7017	2.8733	2.8909	2.9162	2.9193	2.9588	2.9654	2.9606	2.9593	2.9542
	$A_u$	− 1.750	− 1.384	− 1.297	− 1.267	− 1.263	− 1.118	− 1.092	− 1.113	− 1.095	− 0.936
	$R^2_u$	1.0000	1.0000	0.9550	0.9194	0.9226	0.9224	0.9181	0.9912	0.9358	0.9598

$D_w$ and $D_u$ are fractal dimensions of waviness and unevenness, respectively. $A_w$ and $A_u$ are the coefficients during linear correlation for estimating $D_w$ and $D_u$, respectively. $R^2_w$ and $R^2_u$ represent the coefficients of determination during linear correlations for estimating $D_w$ and $D_u$, respectively.

**Table 3:** Fractal dimensions of waviness and unevenness of rock joint sample S3 at varying sample sizes.

Resolution (mm)	Fractal estimation	Sample size ($\mathrm mm \times mm$)									
		$100 \times 100$	$200 \times 200$	$300 \times 300$	$400 \times 400$	$500 \times 500$	$600 \times 600$	$700 \times 700$	$800 \times 800$	$900 \times 900$	$1000 \times 1000$
0.5	$D_w$	2.2477	2.2873	2.2698	2.2469	2.3093	2.3098	2.3084	2.3076	2.2943	2.2830
	$A_w$	− 3.234	− 3.069	− 3.009	− 3.043	− 2.894	− 2.888	− 2.876	− 2.879	− 2.928	− 2.982
	$R^2_w$	0.9916	0.9893	0.9904	0.9967	0.9880	0.9892	0.9913	0.9932	0.9952	0.9953
	$D_u$	2.6727	2.7419	2.7438	2.7682	2.6501	2.7210	2.7333	2.7482	2.7384	2.7793
	$A_u$	− 2.032	− 1.821	− 1.797	− 1.689	− 2.009	− 1.885	− 1.868	− 1.821	− 1.838	− 1.567
	$R^2_u$	0.9855	0.9399	0.9155	0.9636	0.9974	0.9399	0.9450	0.9214	0.9130	0.9378
1.0	$D_w$	2.3202	2.3957	2.3581	2.3260	2.3816	2.3769	2.3761	2.3657	2.3411	2.3428
	$A_w$	− 2.428	− 2.167	− 2.228	− 2.255	− 2.105	− 2.144	− 2.132	− 2.161	− 2.243	− 2.246
	$R^2_w$	1.0000	1.0000	0.9956	0.9992	0.9956	0.9928	0.9954	0.9977	0.9981	0.9989
	$D_u$	2.5722	2.6396	2.8531	2.9117	2.6544	2.9119	2.8671	2.9188	2.8963	2.9262
	$A_u$	− 2.145	− 1.989	− 1.311	− 1.156	− 1.948	− 1.163	− 1.296	− 1.141	− 1.206	− 0.9582
	$R^2_w$	1.0000	0.9959	0.9293	0.9625	0.9969	0.9604	0.9339	0.9932	0.9130	0.9246
2.0	$D_w$	2.3783	2.4380	2.4263	2.4052	2.4282	2.4191	2.4032	2.3904	2.3671	2.3644
	$A_w$	− 1.101	− 1.890	− 1.519	− 1.552	− 1.502	− 1.544	− 1.590	− 1.624	− 1.679	− 1.646
	$R^2_w$	1.0000	0.9934	0.9827	0.9861	0.9923	0.9949	0.9969	0.9988	0.9985	0.9955
	$D_u$	2.7348	2.8765	2.8992	2.9086	2.9207	2.9497	2.9641	2.9613	2.9620	2.8954
	$A_u$	− 1.630	− 1.338	− 1.276	− 1.262	− 1.233	− 1.132	− 1.079	− 1.091	− 1.086	− 1.004
	$R^2_u$	1.0000	1.0000	0.9531	0.9187	0.9175	0.9232	0.9202	0.9870	0.9661	0.9854

$D_w$ and $D_u$ are fractal dimensions of waviness and unevenness, respectively. $A_w$ and $A_u$ are the coefficients during linear correlation for estimating $D_w$ and $D_u$, respectively. $R^2_w$ and $R^2_u$ represent the coefficients of determination during linear correlations for estimating $D_w$ and $D_u$, respectively.

**Figure 10:**
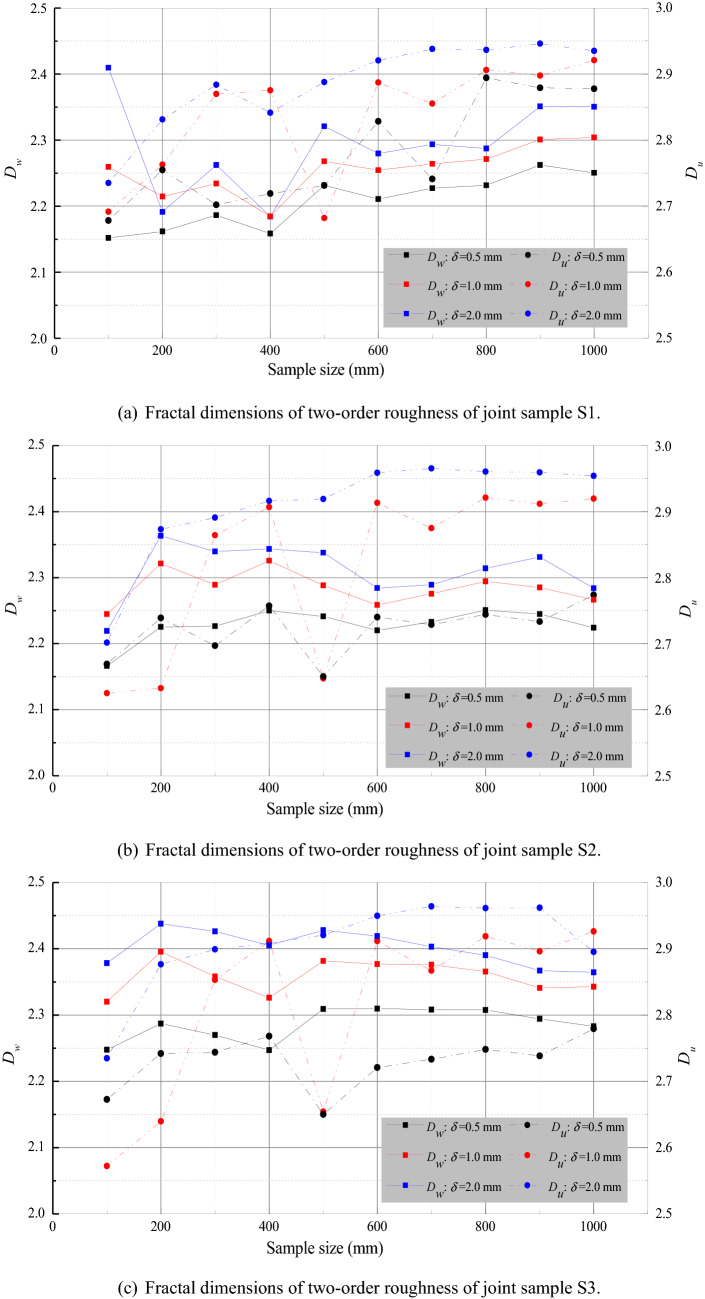
Fractal dimensions of waviness and unevenness of three rock joints of three resolutions at varying sample sizes. $D_w$ and $D_u$ are fractal dimensions of waviness and unevenness, respectively. $\delta$ denotes the resolution.

Figure 10 shows the scale dependence of the fractal dimensions of two-order roughness of the three joint samples at three different resolutions. The fractal dimensions of waviness and evenness of all the three joint samples vary as the sample size increases from 100 mm × 100 mm to 1000 mm × 1000 mm but without universal trends and obvious stationarity thresholds. For the joint sample S1, the fractal dimension of waviness generally decreases as the sample size ascends to 400 mm × 400 mm, after which the fractal value mostly increases as the sample size rises to 1000 mm × 1000 mm. The fractal dimension of unevenness exhibits an increasing trend until the sample size reaches 800 mm × 800 mm, followed by rough level-off. Specifically, the fractal dimension of unevenness is approximately the minimum at the sample size of 100 mm × 100 mm and maximizes at the sample size of 800 mm × 800 mm. As the sample size increases from the minimum to the maximum, the fractal dimension of unevenness increases gradually with several fluctuations.

For the joint sample S2, the fractal dimension of waviness increases as the sample size grows to 200 mm × 200 mm, followed by gradual decrease along with the sample size enlarged to 1000 mm × 1000 mm. The fractal dimension of unevenness generally increases slowly as the sample size grows from 100 mm × 100 mm to 1000 mm × 1000 mm with a few fluctuations. Similar to that of the joint sample S1, the fractal dimension of unevenness of the joint sample S2 generally rises as the sample size grows from 100 mm × 100 mm to 800 mm × 800 mm with main fluctuations at the sample size of 500 mm × 500 mm and remains nearly constant for the sample size larger than 800 mm × 800 mm.

For the joint sample S3, the fractal dimension of waviness decreases gradually when the sample size exceeds 500 mm × 500 mm, before which the fractal value roughly levels off in a narrow range. The fractal dimension of unevenness resembles those of the joint samples S1 and S2. For all the three samples, both the fractal dimensions of waviness and unevenness vary randomly in the range of about 2.1 to 2.5. The variation of the fractal dimension of waviness is less pronounced than that of the unevenness. In terms of digitising resolution, the fractal dimensions of both waviness and unevenness generally decrease as the resolution is enhanced. The mathematical nature of the roughness-length method is responsible for this decrease in fractal dimension along with improved resolution stems. For a rock joint surface, the root mean squares ($RMS_w(\omega )$ and $RMS_u(\omega )$ in Eq. (6)) grow as the digitising resolution is magnified, leading to higher values of the Hurst components ($H_w$ and $H_u$ in Eq. (5)). The fractal dimensions ($D_w$ and $D_u$ in Eq. (3)) are thus decreased correspondingly.

To quantify the variations of fractal dimensions of waviness and unevenness as the sample size is enlarged, the percent error relative to the value of the sample size of 100 mm × 100 mm is calculated:

7 $$\begin{aligned} \delta _i=\frac{|D_i - D_{100}|}{D_{100}} \times 100\% \end{aligned}$$

where $\delta _i$ and $D_i$ represent the percent error and fractal dimension of waviness or unevenness at a sample size between 200 mm × 200 mm to 1000 mm × 1000 mm, respectively. $D_{100}$ is the fractal dimension of waviness or unevenness at the sample size of 100 mm × 100 mm.

**Figure 11:**
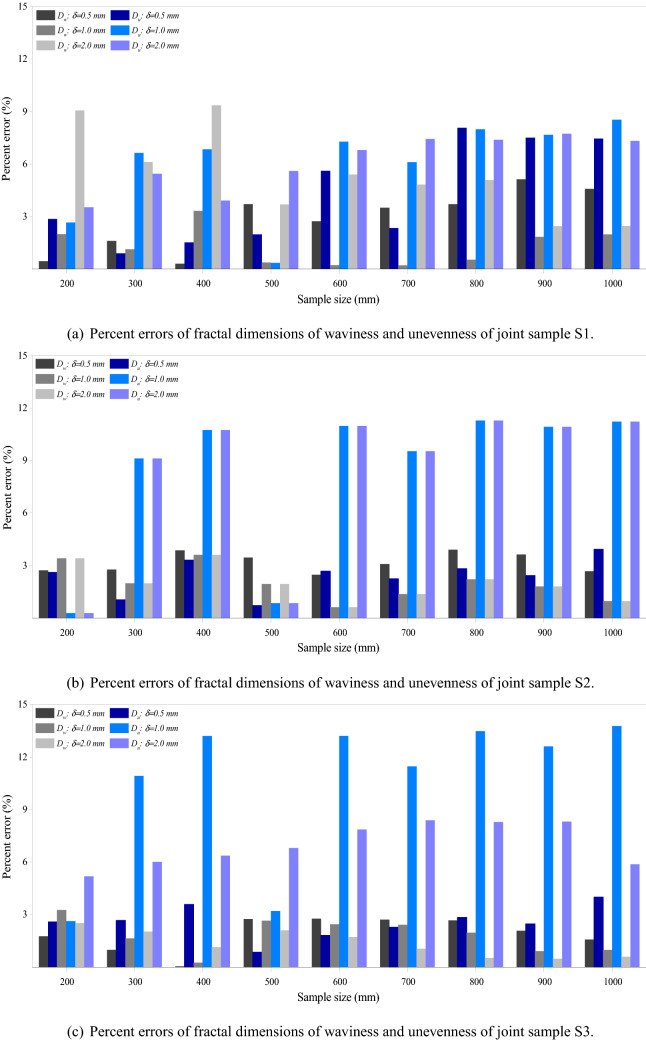
Effect of sample size on the fractal dimensions of waviness and unevenness of three rock joints of three resolutions. Percent errors are relative to the values of window size of 100 mm × 100 mm. $D_w$ and $D_u$ are fractal dimensions of waviness and unevenness. $\delta$ denotes the resolution.

**Figure 12:**
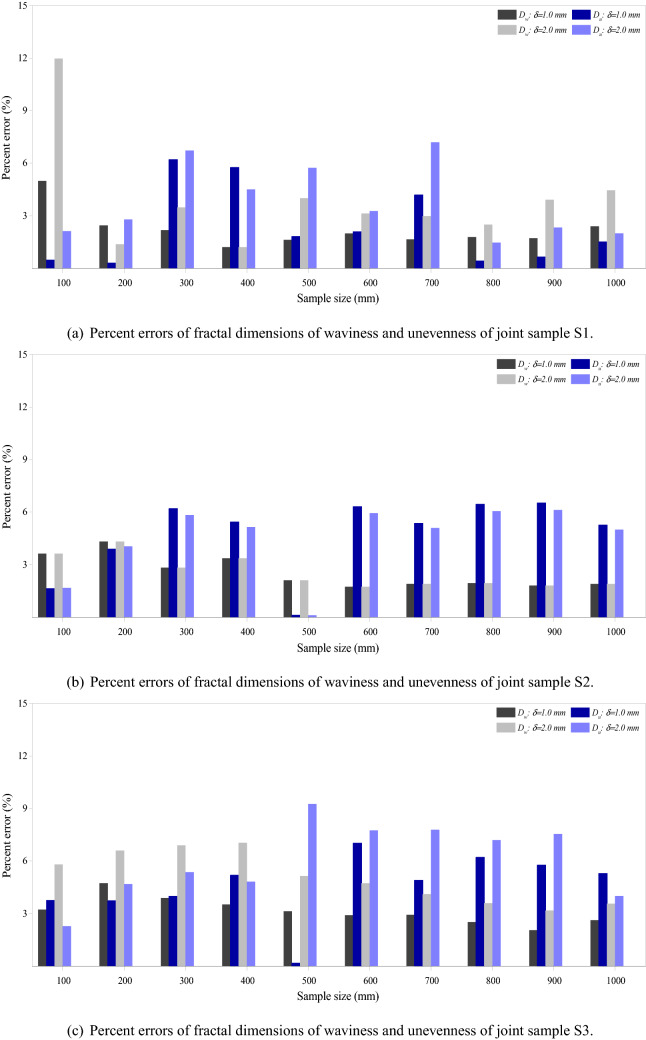
Effect of resolution on the fractal dimensions of waviness and unevenness of three rock joints of varying sample sizes. Percent errors are calculated relative to the values of resolution of 0.5 mm. $D_w$ and $D_u$ are fractal dimensions of waviness and unevenness, respectively. $\delta$ denotes the resolution.

It has been widely documented that sampling interval/solution affects the surface roughness characterization^25,43^. Figure 11 shows the percent errors of fractal dimensions of waviness and unevenness of the three samples at sample sizes from 200 mm × 200 mm to 1000 mm × 1000 mm at three resolutions. Generally, the effect of sample size on the fractal dimension of unevenness is more significant than on that of waviness, particularly for the joint samples S2 and S3 with maximum percent errors around 12% and 14%, respectively. For the joint sample S1, the maximum percent error of unevenness is around 8%, which is slightly lower than that of the waviness at about 10%. The resolution also affects how the fractal dimensions of waviness and unevenness vary as the sample size changes. The variations of the fractal dimensions of waviness and unevenness increase substantially as the resolution changes from 0.5 mm to 2.0 mm, particularly for the joint samples S2 and S3. Figure 12 illustrates the effect of resolution on the percent errors of fractal dimensions of three joint samples of different sample sizes. General comparison of Figs. 11 and 12 suggests that resolution has negligibly less influence on the fractal dimensions of waviness and unevenness than the sample size. In other words, both the sample size and the measurement resolution affect the values of fractal dimensions of the two-order roughness of the joint samples. But the general trend of scale effect of each-order roughness, namely the absence of the stationarity threshold is not changed by the measurement resolution.

## Discussion

Previous studies reported debatable results on the scale dependence of the rock joint roughness Tatone and Grasselli^2^. Since these investigations unanimously using a single fractal dimension did not decompose the surface roughness into different orders, the conflicting outcomes possibly originate from the composition of the scaling of waviness and unevenness. That is, the displayed scaling behaviour of a rock joint roughness is a resultant of the roughness variations of both waviness and unevenness along the varying sample size. For example, the surface roughness of a rock joint possesses an increasing fractal dimension of waviness but a decreasing value of unevenness as the sample size grows likely leads to a reported no scale effect through conventional approaches. On the other hand, the combination of a positive scale effect of waviness and a no scale effect of unevenness may generate an overall positive scale effect.

Based on the fractal characterisation of two large-scale rock joints dimensioned of 1000 mm × 1000 mm and 4000 mm × 4000 mm, respectively, Fardin et al.^9^ and Fardin et al.^10^ claimed that there may exist a stationarity threshold of the scale effect for the surface roughness of a rock joint. Their observations of stationarity limits can be explained as the following. As described clearly by Fardin et al.^9^, the natural rock joint used for replication was almost planar. The minor portion of first-order waviness was further excluded by the original roughness-length method employed for fractal estimation. That is to say, only the second-order unevenness was considered in the scale effect examination. The fractal dimension of unevenness with similar shape probably becomes almost constant as the sampled data arrives at a substantially large volume as the fractal dimension are essentially estimated on the basis of statistical examination. For an instance, the unevenness of the rock joint samples S2 and S3 seemingly share the same stationarity threshold of 800 mm × 800 mm (See Fig. 10 with highly similar results to Fig. 12 in Fardin et al.^9^). Similarly, in the study of Fardin et al.^10^, only the first-order waviness was involved in fractal calculation since the second-order roughness was not captured due to the limited capacity of the low-resolution in-situ 3D laser scanner.

The scale effect of shear strength of rock joints is closely related with the roughness variation at different scales^1,5,44^. Bandis et al.^1^ reported a positive scale effect of joint shear strength, namely, the joint shear strength decreases with increased sample scale mainly due to the same trend of the joint surface roughness rated by *JRC*. On the other hand, Hencher and Richards^44^ showed that the joint shear strength at difference scales were scattered without generally-consistent trend. Our findings on the scale effect of two-order roughness suggest that how the joint shear strength changes along growing scale depends on the changes of waviness and unevenness. Specifically, for a wavy joint with minor unevenness, the shear behaviour and thus the scale effect of shear strength is controlled by the scaling characteristics of the waviness; and this dependance is also valid for the rough joint without noticeable waviness. Due to the composition of waviness and unevenness at different scales, the joint shear strength changes commensurably and exhibits different scale effects as observed by previous studies.

## Conclusions

We examined the scale dependence of two-order roughness of three granite joints with different surface roughness in increasing sample sizes from 100 mm × 100 mm to 1000 mm × 1000 mm through fractal consideration. A high-resolution 3D optical scanner was employed to accurately acquire the surface morphological properties of the three large-scale granite joints in the resolutions of 0.5 mm, 1.0 mm, and 2.0 mm, respectively. The first-order roughness, waviness and second-order roughness, unevenness of a whole joint surface were quantitatively decomposed by selecting the most appropriate sampling interval. The fractal dimension of each-order roughness was calculated using the modified roughness-length approach that quantifies the relationship between fractal dimension and statistical characteristics of the roughness geometry. We found that the fractal parameters of each-order roughness at various window sizes of all the three joint samples were scale-dependent. Nevertheless, there was no obvious stationarity threshold beyond which the fractal dimension of the roughness remains unvaried. Additionally, the measurement resolution has a remarkable influence on the fractal dimensions of both-order roughness. Therefore, when characterising the surface roughness of a rock joint in different scales, the consistency in measurement resolution should be ensured. Existing studies on the scale effect of rock joint roughness reported positive, negative and no scale effects. The conflicting results may be attributed to the combination of the scaling behaviour of waviness and unevenness since previously different-order roughness were examined together through a single fractal dimension without separate treatments.

In rock-engineering practice, the significance of two-order roughness rests on the project type and its boundary constraints. In the low-stress environment where the normal stress acting on the rock joint is low, unevenness mainly dictates the shear behaviour and thus the stability of near-surface underground excavations and surface structures like rock slopes. On the other hand, waviness governs the shear resistance and the stability of rock structures architected in the highly-stressed deep underground. The importance of roughness is assessed by simultaneous consideration of the project type and engineering judgement. Then the waviness and unevenness of a rock joint are characterised at the specific field scale in a consistent resolution with sufficient accuracy. In this study, natural granite joints up to 1000 mm × 1000 mm were sampled for fractal calculation. There is a possibility that the stationarity threshold exceeds 1000 mm × 1000 mm, which is outside of the current observation scope. We will further explore the scale dependence of the two-order roughness of larger rock joints of tens of metres when equipped with a much more powerful scanning system.
